# The Third-Generation Sequencing Challenge: Novel Insights for the Omic Sciences

**DOI:** 10.3390/biom14050568

**Published:** 2024-05-10

**Authors:** Carmela Scarano, Iolanda Veneruso, Rosa Redenta De Simone, Gennaro Di Bonito, Angela Secondino, Valeria D’Argenio

**Affiliations:** 1 Department of Molecular Medicine and Medical Biotechnologies, Federico II University, Via Sergio Pansini 5, 80131 Napoli, Italy; 2 CEINGE-Biotecnologie Avanzate Franco Salvatore, Via G. Salvatore 486, 80145 Napoli, Italy; 3 Department of Human Sciences and Quality of Life Promotion, San Raffaele Open University, Via di Val Cannuta 247, 00166 Roma, Italy

**Keywords:** third-generation sequencing, PacBio, Oxford Nanopore Technologies, genome sequencing, RNA sequencing, epigenetics, metagenomics

## Abstract

The understanding of the human genome has been greatly improved by the advent of next-generation sequencing technologies (NGS). Despite the undeniable advantages responsible for their widespread diffusion, these methods have some constraints, mainly related to short read length and the need for PCR amplification. As a consequence, long-read sequencers, called third-generation sequencing (TGS), have been developed, promising to overcome NGS. Starting from the first prototype, TGS has progressively ameliorated its chemistries by improving both read length and base-calling accuracy, as well as simultaneously reducing the costs/base. Based on these premises, TGS is showing its potential in many fields, including the analysis of difficult-to-sequence genomic regions, structural variations detection, RNA expression profiling, DNA methylation study, and metagenomic analyses. Protocol standardization and the development of easy-to-use pipelines for data analysis will enhance TGS use, also opening the way for their routine applications in diagnostic contexts.

## 1. Introduction

The introduction of innovative and affordable sequencing techniques has determined a revolution in terms of the time and costs of genomic analyses compared with Sanger sequencing, which still remains useful for applications where high throughput is not required. Indeed, so-called second-generation sequencing or next-generation sequencing (NGS), which has been commercially available since 2005, has significantly evolved during the last years in order to provide increased data output and efficiencies, as well as many applications allowing for fast and accurate analyses in different fields [1,2,3,4].

Even if NGS has become a standard tool for many applications in basic biology, and many NGS platforms differ just in sequencing chemistry and/or throughput/sequencing runs, the major issue they share is represented by the generation of short reads, which still is a drawback. Indeed, genomes often contain repeated sequences that are longer than the NGS reads, a condition that may lead to misassemblies, mismapping, and gaps. In addition, while small variants, such as single-nucleotide variations (SNVs) and short indels can be accurately detected using short reads, larger structural variations are more challenging to detect and characterize through these sequencing methods. At the same time, the fact that NGS techniques rely on PCR causes difficulties with regions with high GC% content because they are inefficiently amplified by PCR. In addition, besides possible amplification bias, PCR also removes DNA/RNA modifications, which cannot be detected by NGS directly. Moreover, even if some PCR-free protocols for library preparation are currently available, it has to be mentioned that NGS is a clonal-based method, since the libraries are amplified before the sequencing reactions occur. Finally, specialized bioinformatics tools and complicated postprocessing pipelines are required to manipulate the high-throughput data obtained by the analysis [5,6].

**Figure 1:**
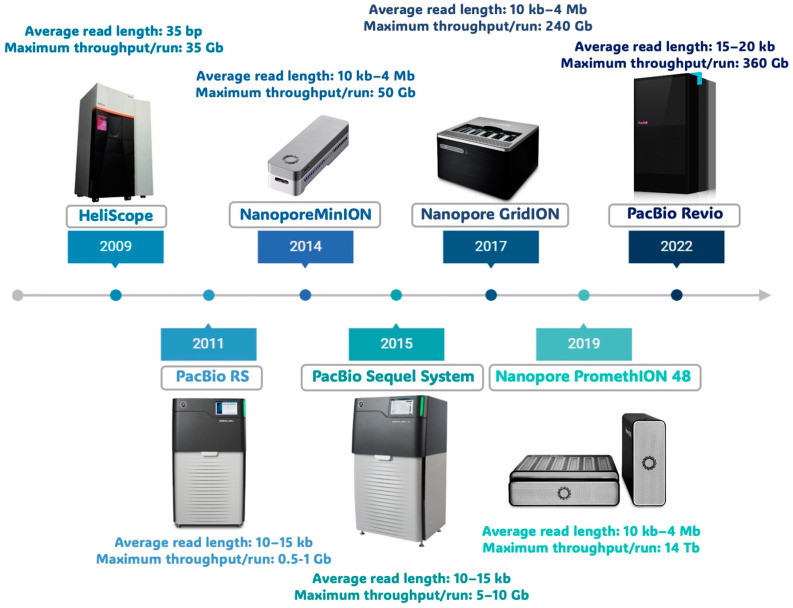
Third-generation sequencing timeline.

In short, even if NGS methods had completely transformed the way we deal with molecular biology nowadays, in recent years, the pressing need to overcome the abovementioned issues made scientists develop a novel methodology that marked the beginning of a new era in sequencing. It is called third-generation sequencing (TGS) and is characterized by improved sequencing chemistry, leading to real-time sequencing and the production of long reads that currently have an average length of more than 10 kb. This is a crucial property aimed at enhancing the quality of genome assembly and the analysis of genomic structures, such as the characterization of large insertions, deletions, translocations, and other structural changes that may exist throughout genome reads. TGS is in general characterized by single-molecule sequencing; this feature makes TGS fundamentally different from clonal-based NGS methods since it enables sequencing DNA or RNA that bypasses the PCR amplification step and, consequently, its potential biases [7]. The first TGS technologies were developed in 2009 with the HeliScope platform launched by Helicos Bioscience, Cambridge, MA, USA (Figure 1).

This single-molecule sequencing method was based on the use of fluorescently labeled nucleotides following a library preparation step [8]. Even if the library protocol overcame PCR amplifications, this method still had critical issues related to high costs and time-consuming procedures; moreover, the sequencing chemistry showed a high error rate and produced very short reads (Figure 1). In the subsequent years, other TGS technologies were developed, launched on the market, and continuously updated to ameliorate their performances in terms of both nucleotide identification accuracy and read length (Figure 1). In 2014, Illumina (San Diego, CA, USA) introduced a library preparation kit for synthetic long reads (SLRs) as an alternative approach, but still based on classical Illumina sequencing chemistry and not linked to the innovative TGS strategies; this methodology uses classical short-read sequencing; therefore, the resulting barcoded reads are assembled locally as they must be derived from the same original large fragment, showing similarities with the shotgun BAC-by-BAC sequencing [7].

Given TGS methodological improvements, their diffusion is increasing as good effectiveness is being demonstrated for different applications. Since it is expected that TGS will become even more used in the near future with their introduction in molecular diagnostic settings, here, we review the current applications and future perspectives of TGS. The main features of the currently available TGS platforms are also described to highlight the pros and cons of each of them.

## 2. Third-Generation Sequencing Methods

As mentioned above, TGS methods were developed in an attempt to overcome NGS limitations, mainly the read lengths and the PCR amplification steps required during library preparation protocols. During the last 10 years, TGS technologies grew up, progressively ameliorating their accuracy and improving both productivity and sequences’ length. Currently, two different TGS platforms, based on very different sequencing chemistries, PacBio Sequencing (Menlo Park, CA, USA) and Oxford Nanopore Technologies (ONT, Oxford, UK), have shown their robustness and are discussed in detail in this article [7,9,10,11,12,13,14,15,16,17].

### 2.1. PacBio Sequencing

**Figure 2:**
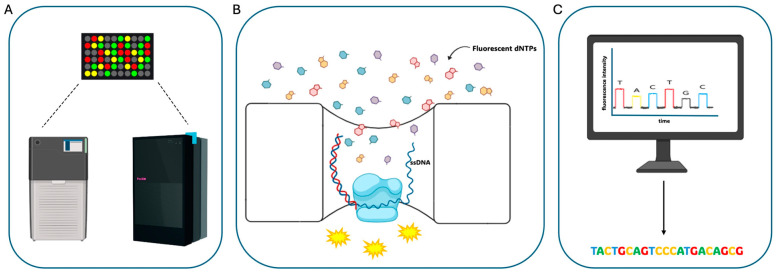
PacBio Sequencing method: The different instruments employ the same chemistry based on the use of a silicon chip, called an SMRT (Single-Molecule, Real-Time) cell, which hosts millions of wells for sequencing reactions (**A**). In each well, a single DNA molecule is immobilized and can be replicated following the injection of fluorescently labeled nucleotides (**B**). Fluorescent signals are registered and used for base-calling (**C**).

The first TGS technology that accomplished the long-reads goal appeared in 2011 when Pacific Biosciences (PacBio) released its PacBio RS sequencer, still characterized by high error rates (~13%) and relatively long read lengths (~1.5 kb) (Figure 1) [9]. Soon after, PacBio improved the first instrument version through a new sequencer, called Sequel System, then followed it up with two additional platforms, namely Sequel II System and Sequel IIe System. The Sequel System Family instruments share common technical features but are characterized by a gradual increase in data output. Indeed, over the years and according to the launch of novel platforms, PacBio improved its maximum output starting from the 500,000 highly accurate reads generated by the Sequel System up to the 4,000,000 reads obtained by the Sequel IIe System, which also makes a difference in terms of computational costs and fast data transfer [10]. Moreover, the recently launched Revio system, by using a high-density sequencing plate and being able to run four independent stages simultaneously, shows a greatly improved sequencing output equivalent to 360 Gb/day with respect to the 24 Gb/day of the Sequel IIe (Figure 2A). This increased throughput is also associated to a simplified workflow for samples preparation, integrated software for base-calling, reads generation and demultiplexing, and increased accuracy (up to 99.95%).

PacBio or Smart Sequencing technology is based on the use of a silicon chip called an SMRT (Single Molecule, Real-Time) cell, which is composed of 8 (the Sequel IIe) or 25 (the Revio) million nanometer-scale wells. Independent of their number, these wells, or zero-mode waveguides (ZMWs), are microscopic chambers where high-fidelity DNA sequencing reactions occur in real time (Figure 2B) [10]. Indeed, circularized DNA fragments are loaded in the SMRT cell and flow within the ZMWs, being immobilized at their bottom. A single DNA molecule is immobilized in each ZMW, so when the labeled nucleotides are added, the DNA polymerase, attached to the DNA during the library preparation procedure, starts to replicate them. Whenever a nucleotide is incorporated in the newly synthesized strand, the fluorescent light is registered and associated with a specific base in order to ensure base-calling accuracy. It has to be noted that the DNA polymerase copies the circularized DNA molecule several times, generating several copies of the same DNA fragments in each ZMW. This process enables one to increase sequencing accuracy since each ZMW produce a consensus sequence by correcting incorporation errors present in a small fraction of copies [11].

However, before the sequencing happens, library preparation is crucial to achieve a pool of circularized DNA fragments required for the subsequent sequencing reactions [10,11]. Library preparation consists of double-strand DNA fragmentation and hairpin adapters ligation onto DNA molecules, thus obtaining circularized constructs, named SMRTbells, on which the DNA polymerase can act. Since an SMRTbell forms a closed circle, after the polymerase replicates one strand of the target dsDNA, it can continue using the adaptor and then the other strand as a template. If the lifetime of the polymerase is long enough, both strands can be sequenced multiple times. As mentioned above, this process is crucial to generate the consensus sequence of multiple subreads in a single ZMW (circular consensus sequence (CCS)), allowing for sequencing with higher accuracy and lower error rate [12].

**Figure 3:**
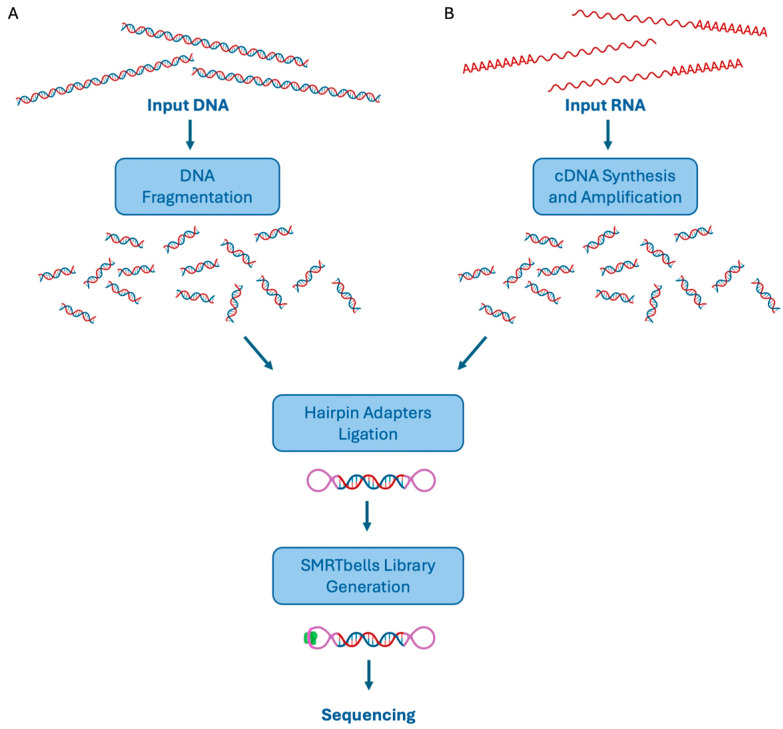
PacBio library preparation workflows: Using genomic DNA as starting material, the first step of the library preparation procedure is represented by DNA fragmentation. Then, DNA fragments are hairpin ligated to obtain an SMRTbell library suitable for polymerase binding and sequencing (**A**). Full-length mRNAs can also be used as an input sample. Indeed, the mRNAs are retro-transcribed, amplified, and hairpin ligated. The obtained SMRTbell library is ready for sequencing (**B**).

To date, several procedures have been developed for both DNA and RNA library preparation, depending on the final aim of the downstream applications [10]. As already stated, the first step of a DNA library preparation protocol is represented by DNA molecule fragmentation; the fragment size is usually about 15–20 kb but, depending in this case on the following application, it can be easily varied by modifying the fragmentation conditions. Indeed, the fragmentation step can be achieved in different ways, ranging from DNA shearing to PCR-based enrichment of the targets of interest. In the latter, it has to be underlined that PCR biases may occur, as for NGS-based procedures; similarly, in the case of small amounts of DNA samples, PCR amplifications may be necessary to obtain enough quantity of starting material [10]. Anyway, the generated double-strand DNA fragments are ligated to universal hairpin adapters, thus obtaining the SMRTbell templates. The advantage of generating these constructs is to obtain, for each DNA fragment, both sense and antisense strands and to allow a high accuracy of CCS generation. Moreover, all the SMRTbells will have the same universal site, allowing for primer binding and sequencing initiation, and the hairpin structure will protect the templates from the exonuclease digestion that is usually used to efficiently remove failed ligation products. After this purification step, the sequencing primer is annealed to the template, allowing the subsequent binding of the DNA polymerase (Figure 3A).

With regard to RNA libraries preparation, the Iso-Seq method allows analyzing full-length transcripts and does not require cDNA fragmentation and subsequent transcript assembly [10]. In particular, cDNA is obtained from total RNA, PCR amplified, and ligated to the hairpin adapters, obtaining the SMRTbells library (Figure 3B) [10]. This procedure can be coupled to a MAS (Multiplexed Arrays)-Seq concatenation method to ligate more cDNA molecules in a longer one, thus increasing the throughput of transcript isoforms sequencing [12].

Regardless of the library preparation protocol used, synthesis reactions are measured within thousands of wells through the incorporation and the detection of fluorophore-labeled dNTPs: it enables the observation of the emitted light that is recorded by a camera in real time; afterward, the signal is translated into nucleotide sequence, a process known as base-calling (Figure 2C) [10,11,12,13]. Once the data from all the ZMWs in an SMRT cell have been produced and registered, primary data are generated as output to be used for downstream analyses. It has to be noticed that when sequencing reactions occur, both the Sequel IIe and the Revio systems are able to register the speed of each nucleotide’s incorporation by the polymerase. This allows one to evaluate the methylation status of each nucleotide, allowing one to achieve simultaneous sequence data and direct methylation detection.

The main benefits of PacBio sequencing technologies are (i) read lengths of 15,000–20,000 bp on average, allowing for easy genome assembly and full-length mRNAs sequencing; (ii) high sequencing accuracy (up to 99.9%); (iii) the possibility to analyze difficult-to-sequence genomic regions, including homopolymers, highly repeated regions, and GC-rich regions; and (iv) direct methylation detection. However, compared with other TGSs, smaller read lengths and higher platform costs may be a drawback [10,11,12,13,14].

### 2.2. Oxford Nanopore Technologies

**Figure 4:**
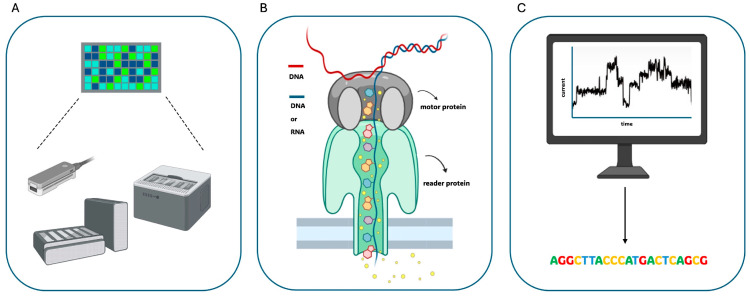
Oxford Nanopore Sequencing method: Several instruments featuring a different throughput are available, all based on the use of nanosensors capable of detecting changes induced by the DNA molecules in the electric current in real time (**A**). Indeed, the flowcell contains thousands of nanopores, each one able to measure the electric current flowing through; so, when a DNA molecule passes inside a pore, it modifies the current according to its sequence (**B**). This typical “squiggle” is used for subsequent base-calling (**C**).

In addition to PacBio’s methodology, TGS technologies saw, in 2014, the development of another approach based on a totally different sequencing method: the nanopore sequencing introduced by Oxford Nanopore Technologies (ONT), which has been successful since its beginning in multiple research fields (Figure 1). This technology, similar to PacBio’s, does not require PCR amplification or any secondary signals in order to achieve the final sequence. However, it does not take a sequencing-by-synthesis approach; instead, it directly identifies the changes in the electric current that are produced in real time. Indeed, the nanopore system is composed of nanosensors through which DNA or RNA penetrate and whose movement is detected across the flow cell. Nanopore flow cells contains a set of microscopic holes, the nanopore, incorporated into an electroresistant membrane (Figure 4A).

The categories of nanopores used in nanopore technology can be divided into solid-state and biological nanopores: the solid-state nanopores, composed of Si_3_N_4_ and SiO_2_, are the most widely used, whereas biological nanopores are usually produced by selected bacteria, such as α-hemolysin pore proteins MspA from *Mycobacterium smegmatis*, Phi29 from *Bacillus subtilis*, and CsgG from *Escherichia coli* [15]. The nanopores are arrayed in microscaffolds to increase their stability: each of them corresponds to their own electrode connected to a channel within the sensor chip, which enables the measure of the electric current that flows through the nanopore. Indeed, a molecule passing through a nanopore disrupts the electric current, producing a characteristic so-called “squiggle” (Figure 4B). The latter is then associated with a specific nucleotide using a base-calling algorithms in order to determine the real-time sequencing of that molecule (Figure 4C) [16].

**Figure 5:**
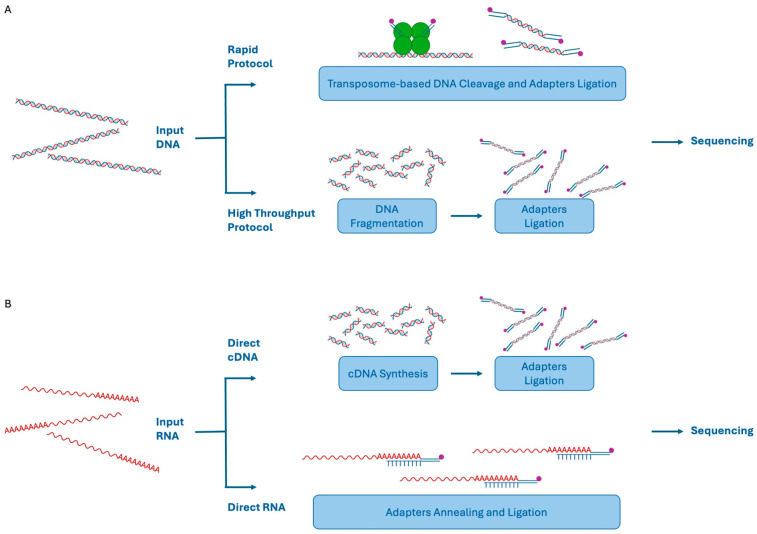
ONT library preparation workflows: DNA libraries can be obtained by a rapid protocol that employs a transposase for both DNA cleavage and adapters ligation or by a high-throughput procedure requiring DNA fragmentation followed by adapters ligation (**A**). RNA libraries can be achieved by cDNA synthesis and adapter ligation or by direct adapter ligation to RNA molecules (**B**).

More in detail, nanopore sequencing requires library preparation, in which DNA fragments are end-repaired, followed by adapter ligation. ONT provides several library preparation procedures both for DNA and RNA, and novel protocols are continuously developed and validated. Briefly, the DNA library preparation can be achieved by using two different strategies: a rapid one and a high-throughput workflow (Figure 5A) [10]. The first one allows fast library preparation by using a transposase complex able to simultaneously cleave the target DNA and ligate to each fragment the adapters required for the subsequent sequencing reactions (Figure 5A). The high-throughput protocol, instead, includes a DNA fragmentation step, fragments ends repair, and adapter ligation (Figure 5A). As for the PacBio protocols, ONT also supports the use of PCR to generate more input DNA in the case of limited sample availability.

With regard to RNA library preparation, unlike the other TGS systems, ONT allows direct cDNA and RNA sequencing [10]. Direct cDNA sequencing is obtained by the reverse transcription of poly A-RNAs, adapters ligation, and sequencing. In the case of low input samples, a cDNA amplification step can be added to the workflow. However, the real innovation in this field is the possibility to direct sequence RNA molecules without the need for retro-transcription. Indeed, the adapters are ligated directly to the mRNA molecules that will flow through the nanopores (Figure 5B).

Once a library is obtained, after a denaturation step (required in the case of double-strand libraries for the unwinding of the dsDNA at the pore), a single-strand molecule enters the channel, helped by a so-called “motor protein”, which directs the passage through the pore. Contextually, it allows the “reader protein” to identify the ionic current alteration, which is distinctive for each nucleotide and generates a unique signature for each base (Figure 4B). However, ONT systems do not identify individual bases since the observable current is determined by short nucleotide sequences; in particular, these sequences are composed of approximately five bases and are called k-mers [10]. All the nanopores present in a single flow cell perform sequencing simultaneously, leading to the generation of a substantial data output within a short period of time; in particular, the available flow cells’ chemistry, named R9, enables >98.3% accuracy per single molecule, and this value has been recently increased up to >99% through the new R10 chemistry. It is important to underline that nanopore technology does not have the possibility of sequencing the same strand multiple times, as with SMRT sequencing. Especially, there are three forms of nanopore sequencing: 1D (one-directional), 2D (two-directional), and 1D^2^. One-directional relies just on one strand of DNA sequencing; 2D consists of a hairpin structure aimed to connect two strands, allowing the sequencing of the first strand, immediately followed by the second one. However, to improve read accuracy, alternative strategies have been developed to sequence the second strand only when the first one has already finished passing through the pore, without the need to physically bind the two strands of DNA together through the hairpins; to this aim, the “1D^2^” system has been introduced [6,10,17].

ONT released its first prototype of nanopore sequencer in 2014, the pocket-sized and portable MinION, while the updated platform PromethION was released the following year with an improved throughput (Figure 1). As discussed above, the reaction system for nanopore sequencing is carried out in a flow cell, in which two ionic solution-filled compartments are separated by membranes containing either 2048 (MinION, generating up to 50 Gb/sequencing run) or 12,000 (PromethION, generating up to 290 Gb/sequencing run) nanopores. Specifically, two versions of PromethION, namely 24 and 48, integrate 24 and 48 independent flow cells, respectively, in order to increase productivity by simultaneously using several flow cells. In addition, the PromethION 2 (P2) is a small device allowing for the analysis of up to two PromethION flow cells and has been just released for laboratories with a smaller sample processing number (Figure 4A). All these systems are based on the same sequencing technology but with a scalable throughput. While the PromethION system could output up to 7.6 Tb data (with a theoretical maximum of 15 Tb), the MinION could only generate 50 Gb within 72 h. This feature makes the PromethION a serious competitor for Illumina’s HiSeq X Ten, which generates a theoretical maximum output of 16–18 Tb/run. In early 2017, ONT also released the GridION X5, which can hold up to five MinION flow cells and can generate up to 240 Gb of data per run. ONT also announced the release of the SmidgION, which is even smaller than a MinION and can be controlled by a smartphone. A drawback shared by all the ONT instruments is still their high error rate [7,10,18].

The main features of the two discussed TGS technologies, PacBio and ONT, are summarized in Table 1 and compared with those of Illumina, that is, the most used NGS.

### 2.3. TGS Data Analysis

Typical of both PacBio and ONT is the huge amount of data generated by these kinds of massive parallel sequencing technologies; moreover, both of them provide lower per read accuracy than short-read sequencing. They share the same Hierarchical Data Format 5 (HDF5) for the data storage: while PacBio uses the h5 format, ONT adopts the FAST5 file format. In PacBio’s h5 files, the translation into nucleotide sequence follows the Circular Consensus Sequencing (CCS) workflow, producing the so-called “HiFi” reads, whose quality is heavily dependent on the number of times the fragment is read. Nanopore base-calling is itself more complex than the PacBio one; nevertheless, its quality is independent of the length of the DNA fragment, since read quality depends on achieving the optimal translocation speed of the nucleic acid through the pore, which typically decreases in the late stages of sequencing runs, negatively affecting the quality itself [19]. Each read produced by one of the ONT channel devices integrates algorithms producing FAST5 or FASTQ files.

For both PacBio and Nanopore, the base-called files have been improved in order to reduce the error rate, so much so that these values are decreased to 1% and 5%, respectively, thus allowing the final resulting reads to become proper for downstream analyses, such as base modification detection, transcriptomics studies, structural variation identification, and phylogenetic classification [10].

Indeed, to obtain biological information, these raw data need to be specifically processed, analyzed, and, finally, interpreted. Over the years, several bioinformatic approaches have been developed for NGS data, allowing for their use in several contexts. However, the main constraint is that these tools have been developed and optimized for short-read management. This means that specific bioinformatic pipelines, ranging from read quality controls to downstream applications, are required for long reads. If we consider the huge number of reads that is produced/day, it is feasible to suppose that this aspect has the potential to become the main bottleneck for TGS diffusion and use. To avoid this, great efforts are being made toward the development of software able to handle long-read sequencing data. For example, LongQC [20] and NanoPack [21] are two quality control tools specifically designed for PacBio and ONT sequences. Similarly, assembly algorithms and specific tools for the different downstream applications are becoming available, and their optimization will progressively facilitate TGS diffusion. However, their detailed discussion is beyond the scope of this review; please refer to other works that have further explored these aspects [7,10,17,19].

## 3. Third-Generation Sequencing Applications

**Figure 6:**
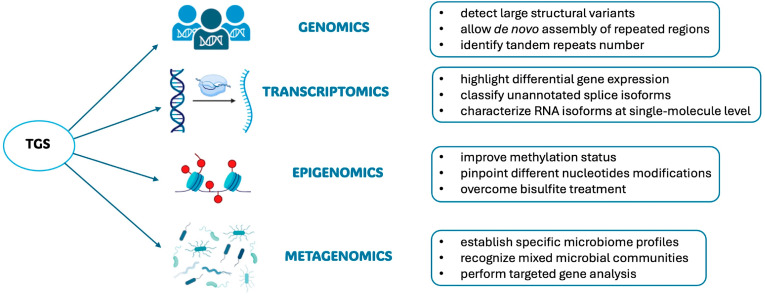
Third-generation sequencing applications and usefulness in different omic fields.

By sequencing thousands of bases with progressively greater accuracy and reducing costs, long-read technologies are becoming even more useful in resolving traditionally difficult-to-map genes or genome regions, such as highly repetitive elements, identifying co-inherited alleles, or getting information about haplotypes, as well as identifying de novo mutations and generating long reads for de novo assembly [5]. Moreover, TGS is making a revolution in omics research, allowing new discoveries in many fields of application, as is reviewed in the next sections (Figure 6).

### 3.1. Genome Sequencing

Even if NGS technologies have allowed the decoding of the human genome in a short time and with lower costs, promoting the use of these techniques in the diagnostic routine of genetic diseases [3,22], they still present some limitations.

As mentioned above, one of NGS’s limits is the difficulty of sequencing highly repeated portions of the genome. These regions represent about 50% of our genome and include transposons, pseudogenes, repeats of a certain number of nucleotides, duplications of long DNA sequences, and tandem repeats, located in specific chromosomal regions (centromeres, telomeres, and short arms of acrocentric chromosomes) [23]. NGS is only able to sequence short reads, which are then overlapped, thus making the de novo assembly of repeated regions challenging [24]. As a consequence, none of the human chromosomes has been fully sequenced by NGS, but each of them contains gaps to be filled [7]. Moreover, this aspect is linked to a further NGS limitation, i.e., the problematic identification of large structural variants. Since structural variants are involved in many human pathologies, their identification is crucial [25]. Finally, the need for PCR amplification during the library preparation step can lead to errors in nucleotide insertion by the polymerase, as well as difficulties in the amplification of GC-rich regions and microsatellites [7]. The advent of TGS is overcoming these limits currently impairing our ability to study the human genome and identify genetic variations related to human disease onset.

In this context, PacBio’s methodology has been tested in clinical research settings, for example, to verify its ability with genomic repeated regions. Alterations of tandem repeat number are involved in many human diseases, such as Fragile X syndrome [26]. In the latter, the trinucleotide CGG repeats, present in the 5′ UTR of the *FMR1* gene, may be present in variable numbers, even greater than 200 repeats. So, this region’s sequencing could be problematic due to its excessive length and the high presence of GC nucleotides. Loomis et al. were able to sequence this region in a single molecule using PacBio real-time sequencing. Interestingly, they showed that 750 CGG repeats could be accurately sequenced and proposed this approach for the screening of both the affected patients and the premutation carriers [27]. Starting from this evidence, many nucleotide repeats involved in other human diseases have been sequenced using the same methodology. McFarland and colleagues used PacBio to study the molecular bases of spinocerebellar ataxia type 10 (SCA10), a neurodegenerative disorder caused by the repeats of the “ATTCT” sequence in the intron 9 of the *Ataxin 10* gene. They were able to identify the number of tandem repeats in three patients with different disease phenotypes using a single molecule that covered the entire expansion [28]. Höijer et al. used the same approach to analyze some unstable CAG trinucleotide repeats involved in the onset of Huntington’s disease [29].

The PacBio sequencing method has also been used to identify other kinds of pathogenic genomic variants. Melas et al. used long-read sequencing to study a family affected by synpolidactyly 1 (SPD) and revealed a 27 bp duplication in a polyalanine stretch in the exon 1 of the *HOXD13* gene, a transcription factor involved in morphogenesis. This alteration was not detected by Illumina sequencing, probably due to the high content of GC nucleotides in alanine encoding codons. A similar alteration, a 21 bp polyalanine expansion, was identified in the same position in another unrelated affected family, thus suggesting that the expansion of this polyalanine tract may be the underlying genetic mechanism of sinpolidactyly in these two families [30]. Borras et al. sequenced the entire genome of 19 patients with autosomal dominant polycystic kidney disease and suggested this approach as a valid one for the identification of pathogenic *PDK1* variants, avoiding the interferences due to *PDK1* pseudogenes [31]. Hiatt et al. analyzed six family trios affected by a neurodevelopmental disorder and without a genetic diagnosis, even after NGS analyses, and identified a pathogenic variant in 2/6 probands [32]. Similarly, Pauper et al. studied five trios with unsolved intellectual disability and identified potential pathogenetic variants [33]. Mehinovic et al. analyzed a family in which two out of the three children were affected by autism and epilepsy episodes and identified a de novo missense variant in the *KCNC2* gene potentially related to the familial phenotype [34]. Recently, a growing amount of evidence has supported PacBio sequencing as an affordable tool for the genetic diagnosis of thalassemia, especially in the presence of rare variants (mainly deletions) or genetic recombination [35]. In this context, Liang et al. developed a PacBio-based comprehensive analysis tool for thalassemia, the CATSA, and used it to analyze 1759 samples, showing its ability to identify common and rare variants, thus increasing diagnostic sensitivity [36]. Finally, PacBio technology has also been used for targeted long-range DNA sequencing. In a recently published paper, this approach was used to analyze a patient with X-linked nephrogenic diabetes insipidus and identify a novel deletion of the *AVPR2* gene [37].

Similarly, ONT has also been proven to identify genetic variants related to human diseases. Mitsuhashi et al. used it to successfully analyze and quantify the number of repeats of the *D4Z4* array on chromosome 4 that, when reduced, is associated with the onset of facioscapulohumeral dystrophy (FSHD) and, due to the presence of repeats and high CG content, is difficult to sequence with NGS [38]. Cretu Stancu et al. sequenced the whole genome of two patients with genetic defects derived from chromotripsis processes and detected de novo structural variations (SVs), including the identification of their chromosomal breakpoints, that were not found by Illumina technology [39]. Leija-Salazar et al., by sequencing the *GBA* gene, whose mutations cause the Gaucher disease, showed that the ONT method allows obtaining the entire gene sequence, avoiding data contamination with its pseudogenes [40]. Bruels et al. used ONT sequencing to analyze a cohort of 12 unsolved individuals from 10 independent families with suspected muscular dystrophy and were able to identify both *DMD* large structural variants and single-nucleotide variants, as well as a *LAMA2* 3.6 Mb duplication [41]. In the context of dystrophy diagnosis, ONT has also been used as a prenatal tool to sequence the whole genome of pregnant women with a DMD duplication, contributing to the identification of its precise breakpoints [42]. Yu et al. used ONT whole-genome sequencing to analyze patients affected by oculopharyngodistal myopathy (OPDM) types 3 and 4, which remained unsolved after NGS, and were able to identify a heterozygous GGC repeat in the *NOTCH2NLC* gene [43] and a heterozygous CCG repeat in the upstream region of the *RILP1* gene [44]. Besides whole-genome sequencing, ONT has also proven its efficacy for targeted applications. Indeed, a targeted long read was used for the analysis of a retinoblastoma patient, allowing the discovery of rare variants and haplotype analysis [45].

In addition to all these examples of successful application in the identification of disease-causing variants, TGS has also been used to resolve assembly gaps and fix some errors present in reference human genomes. Indeed, the Telomere-to-Telomere (T2T) Consortium employed both PacBio and ONT obtains a complete genome reference, the T2T-CHM13, including all centromeric regions and resulting in more accuracy than the GRCh38 [46].

### 3.2. RNA Sequencing

RNA sequence analyses allow one to evaluate gene expression and identify mechanisms that may be involved in their regulation; as a consequence, NGS has been widely used in this context [47,48,49]. However, although NGS platforms have the advantage of sequencing at competitive costs and with a great sequencing depth, these methodologies have some limitations in RNA sequencing applications [50]. Indeed, the reverse transcription of RNA into cDNA and the fragmentation of the targets into short reads cause a loss of information from the native full-length transcript [51]. Thus, TGS has emerged as a potential novel strategy, since it offers several advantages: (i) smaller amount of starting material, (ii) longer sequence reads length, (iii) less time to results, (iv) higher output, and (v) competitive costs [52,53]. Both PacBio and ONT technologies have been used in this context, and several pipelines have been built in order to achieve different outcomes, and many others are emerging to ameliorate the previous one.

Sharon et al., using PacBio for human transcriptome analysis, highlighted four important results: (i) PacBio gave the possibility to detect RNA isoforms at single-molecule level, without amplification and fragmentation; (ii) the quality of the synthesized cDNA was important for the right detection of all splice sites; (iii) unannotated splice isoforms were found (more than 14.5%); and (iv) unannotated intron structures were discovered [54]. Kono et al. used the ONT full-length cDNA sequencing analysis to successfully identify *DSCAM1* (Down syndrome cell adhesion molecule 1) isoforms [55]. Workman et al. showed how the use of ONT long-reads allows for the identification of allele-specific expression (ASE), a feature that is difficult to recognize by using short reads because heterozygous variants are rare and may not occur in the hundreds of nucleotides sequenced by NGS [56].

One of the limits of RNA-Seq through NGS is the necessity, during the library preparation, to convert RNA molecules into cDNA; this kind of procedure eliminates all RNA modifications and may also introduce possible biases or misamplification. In this context, Zhao et al. showed how ONT direct RNA library construction workflow can generate RNA–DNA hybrids, allowing for the direct sequencing of super-long RNA molecules [50]. This procedure allows the detection of RNA modifications, both in position and nature, such as NAD-capped RNA, the second structure of mRNA, RNA modification, like N6-methyladenosine, 5-methylcytidine, 5-hydroxylmethylcytidine, and others [55,57,58]. ONT direct RNA sequencing is also important for the analysis of RNA secondary structures, like rG4 [57]. Aw et al., by coupling chemical modifications with ONT direct RNA sequencing, developed a custom pipeline, the PORE-cupine, able to identify structural transcriptomic patterns [58]. Additionally, the previous high-throughput methods for the analysis of RNA modifications, through the use of short-read sequencing technology, require an enrichment during the library preparation that alters and limits real quantification. ONT overcame these limitations and also allows the detection of 3′ poly(A) tail length, base modifications, and transcript haplotypes [56].

With regard to gene expression quantification and differential expression analysis, NGS-based methods have been widely used in this field, even if their short reads hamper the possibility of discovering and quantifying transcript isoforms that may not be fully covered due to their length. This may be overcome by TGS, a limitation being represented by the availability of proper computational methods. Actually, several bioinformatic tools have been developed for TGS-based gene expression quantification, as reviewed elsewhere [19]. Irrespective of their differences, all of them require accurate gene isoform annotation; next, the quantification step can be performed at the gene level or at the transcript level, followed by differential expression analysis [19]. Despite the increasing use of TGS for transcriptomic studies, performance comparison of these workflows with respect to NGS-based protocols is challenging. The Long-read RNA-Seq Genome Annotation Assessment Project (LRGASP) Consortium aimed to answer this question. Indeed, they analyzed ONT, PacBio, and Illumina RNA-Seq data using different bioinformatic tools and were able to give some recommendations to improve the use of TGS for transcript isoform detection, identification, and quantification [59].

Direct RNA sequencing is also important for viral genomes, since they are featured by multiple reading frames, antisense locations, inefficient termination signals, and complex splice forms [55]. Indeed, Li et al. used ONT to sequence and characterize the SARS-CoV-2 genome from clinical specimens [60].

Finally, it has to be mentioned that, as discussed above, TGS allows one to characterize several RNA features that were not detectable before; this required the development of several specific bioinformatic algorithms. In this context, an accurate review from Xie et al. summarized the bioinformatic tools available for ONT-based RNA data analysis and underlined the possibility of using some bioinformatic tools, like EpiNano, ELIGOS, and nanom6A, for an accurate analysis of RNA modifications [57].

### 3.3. Epigenetics

The term “epigenetics” refers to any modification that happens in gene expression independently from the primary DNA sequence [61]. A fundamental epigenetic mechanism in eukaryotic organisms is DNA methylation. This reversible process is responsible for the transcriptional regulation of many genes and creates a dynamic methylation pattern depending on the different stages of development. Chemically, the most common modification consists in the covalent addition of a methyl group to the 5-carbon of cytosine, resulting in 5-methylcytosines, which are mainly clustered in the “CpG islands”. These areas are characterized by the high-density repetition of the C-G dinucleotide (G + C > 55%) and are located in the promoter region of several genes, mostly in a methylated state [62].

Although DNA methylation has a key role in physiological pathways, such as imprinting and X chromosome inactivation, relevant evidence proved that alterations in these patterns could result in pathological conditions, for instance, loss-of-imprinting syndromes, autoimmune diseases, neurological syndromes, and several cancer types [62,63]. Cancer cells, in particular, show a peculiar epigenetic organization characterized by global genome hypomethylation and the hypermethylation of specific CpGs. These features may have a role in promoting cancer; indeed, the genome hypomethylation may cause chromosomal instability [64] and could also determine the aberrant activation of specific oncogenes when occurring in their promoter regions [65]. Promoter hypermethylation, instead, causes the silencing of many tumor-suppressor genes and is another mechanism involved in carcinogenesis [66].

In view of the multiple implications of aberrant DNA methylation, several strategies to analyze it have been developed. To date, the gold standard in epigenome analysis has been represented by bisulfite sequencing (BS-seq) using NGS platforms [67,68]. In this approach, prior to sequencing, genomic DNA undergoes a sodium bisulfite treatment where unmethylated cytosines are deaminated to uracil leaving the methylated ones intact. The subsequent PCR amplification causes the uracil conversion to thymine, allowing the subsequent short fragments sequencing and assembly to a reference genome [69]. Despite the undisputed NGS advantages, including the possibility to obtain quantitative sequencing of the CpGs across the whole genome and the great accuracy due to high throughput [70], this approach shows some drawbacks in methylation analysis. Firstly, during sample preparation, DNA could go through a massive degradation resulting from the harsh chemical conditions required to obtain a complete bisulfite modified DNA [71]. In addition, bisulfite treatment is unable to differentiate 5-Methyl cytosine from its oxidative form 5-Hydroxymethyl cytosine, whose biological role still remains unclear. Finally, the high content in GC could interfere with the PCR amplification step and could also add a new layer of complexity to an already highly fragmented assembly due to the short reads [72].

To overcome some of these limitations, an active research area is represented by the use of TGS. These methods allow a more accurate assembly of the methylated regions through sequencing single-molecule long reads of approximately 10–16 kilobases (kb) [11]. Moreover, these sequencing methods do not require a bisulfite treatment nor a PCR amplification step, preventing both damage and amplification biases [67].

Indeed, in PacBio real-time sequencing, every nucleotide or modified nucleotide addition generates a pulse characterized by a distinct pulse width and interpulse duration (IPD) that could affect the DNA polymerase kinetics allowing the discrimination of modified nucleotides, such as N6 Methyl-Adenine in bacteria but also Methylcytosine and Hydroxy-Methylcytosine, although with minor accuracy [71]. One major drawback of this technique lies in its high but randomly spread error rate (approximately 10–15%), which can be decreased by using Circular Consensus Sequencing (CCS) as a template [73].

ONT has also been used for methylation analysis [74]. Wallace et al. were able to recognize different nucleotide modifications, including 5 Methyl Cytosine, suggesting that ONT may be a powerful approach for investigating methylation status [75]. In addition, the correct methylation status could be efficiently analyzed, improving the accuracy of base-calling by coupling the pore with a DNA polymerase (DNAP) as motor protein [76,77]. Specifically, an M2MspA pore linked to a phi29 DNAP was used by Wescoe and colleagues to distinguish modified cytosines, including 5-carboxylcytosine(caC) and 5-formylcytosine (fC), which are produced during enzymatic conversion of 5 hmC to cytosine [78].

Furthermore, targeted approaches have been implemented to TGS methods to enable long-read deep sequencing at an affordable cost [79]. Indeed, a selected DNA region can be targeted by the RNA-guided endonuclease activity of Cas9. Nanopore Cas9-targeted sequencing (nCATS) allows the simultaneous assessment of both methylation and the mutations of targeted regions, as reported in the study of clinical-glioma-affected patients conducted by Wongsurawat and colleagues [80].

### 3.4. Metagenomics

Metagenomics is a branch of genetics that focuses on the study of microbial communities living in natural ecosystems in symbiosis with humans and other animals by analyzing the whole microbial genetic material in the collected samples [81].

Since Carl Woese and George E. Fox discovered the importance of the rRNA 16S gene in the differentiation of microbial taxa in 1990 [82], the sequencing of the 16S gene’s hypervariable regions (V1–V6) has been the most accepted method used to characterize and classify microbial communities [83]. Although NGS has been widely used in the last years for this aim due to its ability to provide a huge number of short reads (<500 bps) with a high sequence accuracy (about 99%), it is limited in providing an accurate phylogenetic resolution, especially at the genus level, because of the highly conserved nature of the 16S gene and NGS’s inability to sequence the whole gene [84]. Thus, the possibility of TGS-based technologies to sequence long molecules of DNA and RNA without the need for fragmentation or complementary synthesis made them suitable for metagenomic analyses [85].

Wagner et al., by analyzing the vaginal microbiome of 11 samples using both NGS and PacBio, found similar error rates, even if some discrepancies in taxonomic assignment were identified [86]. Earl et al. used a PacBio-based pipeline for 16S rRNA gene sequencing and were able to increase both taxonomic and phylogenetic resolution [87]. Another comparative analysis revealed that the low sequencing accuracy of PacBio may impair its taxonomic resolution in the study of human gut microbiota [88]. Nevertheless, PacBio sequencing has been successfully used in several metagenomic studies and was able, for example, to identify the microbiome profiles of heavy-metal-contaminated soils [89], analyze extrachromosomal genetic elements (mainly plasmids) in the human gut [90], characterize the airway microbiome of chronic obstructive pulmonary disease patients [91], identify low-biomass human gut phageomes [92], and highlight the association between the indoor microbiome and nasal/oral humans microbiome [93]. A very recent paper by Eisenhofer and colleagues compared shotgun metagenomics carried out with both NGS and PacBio technologies and found that even if PacBio ameliorates the quality of bacterial genome assembly, it is more expensive and needs a higher sequencing coverage with respect to short reads, thus suggesting that the most optimal strategy depends on the aims of each specific project [94].

ONT has also been used for metagenomic purposes. In 2016, a pilot study by Edwards et al. carried out one of the first workflows for nanopore-based shotgun metagenomic sequencing by analyzing the microbial communities of different extreme soil samples: the cryoconite holes upon Svalbard glaciers, the Greenland Ice Sheet, and the Austrian Alps [95]. Next, Xiao et al. evaluated the ONT near-full-length 16S rRNA gene sequencing by using two pure-culture samples of *E. coli* and *P. flurenscens* and a low-diversity environmental sample obtained from hydraulically fractured produced water and highlighted the great potential of this approach to analyze microbial communities and also to identify poorly represented bacteria in mixed microbial communities [96]. Shin et al. compared nanopore and Illumina performances in analyzing the mouse gut microbiota and showed that the two strategies obtained similar taxonomic resolutions, except at the species level, where ONT resulted in more accuracy [97]. Similarly, Benítez-Páez et al., testing the ability of ONT in taxonomic identification of mock communities, were able to assign reads down to the species level and to evaluate their relative abundances [98]. ONT’s sensitivity in identifying less represented organisms in a community has also been demonstrated by Brown et al., who used this approach to analyze mock samples obtained by mixing different amounts of bacteria [99]. Mitsuhashi et al. used ONT to analyze a mock bacterial community that contained equimolar 16S rDNAs and a pleural effusion from a patient with empyema and were able not only to identify all 20 bacterial species present in the mock community but also to detect bacterial pathogens in the effusion [100]. Yang et al. used ONT metagenomics to analyze clinical respiratory specimens and assessed its utility in severe pneumonia diagnosis [101]. Ibironke et al. used the same approach to study the bacterial composition of lung, throat, mouth, and nose from five patients and were able to assert the differences between these biological niches [102]. Taylor et al. compared ONT sequencing with metatranscriptomics and amplicon-based sequencing to profile colorectal tumor tissue microbiome and showed the potential of this approach [103]. Similar results were also obtained by Yoshiyuki M. et al. who, by comparing ONT full-length 16S rRNA gene with NGS amplicon sequencing, assessed the advantages of using long reads for microbial identification [104]. Recently, Chen et al. used ONT to obtain the oral microbiome profile of periodontitis patients and identified the increased abundance of the *Lactobacillus zeae*, thus suggesting its pathogenic role [105].

Although TGS has demonstrated high efficacy in microbial characterization and taxonomic profiling, all these studies are afflicted by its main limitations: relatively higher error rates compared with other platforms, which penalize the accuracy of identification, especially at species and variants levels, as well as the lack for bioinformatic tools customized for TGS data. In recent years, several efforts have been made to reduce TGS error rate. Moreover, specific bioinformatic pipelines for long-read-based metagenomics have been designed and tested, as reviewed elsewhere [85]. Thus, TGS is expected to play a crucial role in this field in the near future.

### 3.5. TGS in Single-Cell Multiomics

Single-cell genomics employs omics techniques, such as single-cell DNA sequencing and single-cell RNA sequencing, to identify genetic variants and nucleotide modifications, as well as analyze gene expression and its regulatory mechanisms at the single-cell level [106]. Thus, single-cell analyses promise to increase our knowledge of the molecular mechanisms underlying disease onset and to advance in an even more personalized approach.

TGS-based methods are also showing their potentialities in this field; indeed, their long reads promise to overcome NGS limitations, especially in the study of structural variants and in the identification of transcript isoforms and extrachromosomal circular DNAs. In particular, Fan et al. developed a TGS-based strategy, the SMOOTH-seq (single-molecule real-time sequencing of long fragments amplified through transposon insertion), for single-cell whole-genome sequencing and showed that their approach was able to detect both structural variants and extrachromosomal circular DNAs in individual cells [107]. Next, the SMOOTH-seq was improved by Chang and colleagues who developed and validated a TGS-based single-cell multiomics approach to analyze both the genome and the transcriptome and demonstrated its feasibility for the study and monitoring of tumor samples [108]. Considering that the number of studies in this field is rapidly growing up, TGS-based single-cell multiomics will become an even more used approach, especially in the study of cancer cells.

## 4. Current Limitations and Future Perspectives of TGS

As expanded on in the previous sections, long-read-based sequencing is showing great potential in several fields. Indeed, it improves read assembly, transcript isoform identification and quantification, structural variants detection, nucleotide modification study, and so on. These features, associated with a progressive reduction in costs and improvements in sequencing accuracy, are prompting TGS diffusion. However, some limitations still occur.

First, instruments and reagent costs may not be affordable for all laboratories. In this context, ONT provides a range of platforms that may partially resolve this issue, even if the smaller instruments have reduced throughput and may not be useful for all downstream applications. In addition, it should be taken into account that the costs of reagents are not so high considering that different analyses (such as variants detection and methylation) can be carried out in a unique assay and that targeted strategies allow to contain the expense [109].

Next, bioinformatic requirements may be a bottleneck. Of course, the huge amount of data obtained with these technologies requires the development of additional bioinformatics tools, algorithms, and specific pipelines necessary for analyzing the insufficient TGS datasets, which still represents a challenging task. Moreover, bioinformatic infrastructure and servers are required for data management and storage.

Once these issues are fixed, together with ameliorated technical features, as well as protocol standardization and integration, TGS may become a routinely used application. In particular, long-read analyses will be useful in a clinical context to improve the diagnostic sensitivity of the currently used molecular tests, especially for the study of structural variants and nucleotide repeats. Moreover, multiomics integration, incorporating different omics data, will contribute to clarifying the correlations between genomic features and their phenotypic expression, moving toward precision health and personalized medicine. TGS-based approaches are showing their potentialities in achieving multiomics data, as well as at the single-cell level. Thus, this field may be largely developed in the near future.

## 5. Concluding Remarks

The studies cited so far have been carried out in the context of clinical research, and their number is exponentially growing, showing TGS potentialities in the identification of disease-causing genomic variants. Taken together, this evidence strengthens the hypothesis that long-read sequencing can be a valid tool in clarifying the etiology of rare genetic diseases in patients whose diagnosis is unknown. As a consequence, TGS could be implemented in the future in the diagnostic routine of human diseases. To this aim, TGS needs to be approved by the International Organization for Standardization (ISO). In addition, cost-effective and unified workflows between laboratories need to be developed. Finally, bioinformatic analysis tools suitable for clinical purposes are also needed, as those currently in use are only appropriate for research studies [110]. The possibility to improve our understanding of the molecular bases of human diseases by efficiently analyzing RNA expression, DNA methylation status, and microbial communities at improved resolution represents additional advantages that will prompt TGS diffusion in the next years.

## Figures and Tables

**Table 1:** PacBio and ONT features comparison. Illumina characteristics are also reported to highlight TGS differences with respect to the widely used NGS technology.

	Third-Generation Sequencing Technologies		Next-Generation Sequencing
Features	PacBio	ONT	Illumina
Sequencing Chemistry	SMRT	Nanopore-based	Sequencing by synthesis
Average reads length	15–20 kb	10 kb–4 Mb	2 × 300 bp ^3^
Base-calling accuracy	up to 99.95% ^1^	99.9%	99.9%
Maximum throughput/run	360 Gb ^1^	290 Gb ^2^	8 Tb ^4^
Cost per Gb *	65–200 $	22–90 $	12–27 $
Complex genomic regions (GC-rich, homopolymers) analysis	Yes	Yes	No
Direct methylation detection	Yes	Yes	No
Pros	Long reads High accuracy Allows direct cDNA analysis Allows direct methylation and other DNA modifications analysis	Very long reads Allows direct RNA analysis Allows direct methylation and other DNA modifications analysis Availability of portable sequencers	High accuracy High sensitivity High multiplexing capacity High versatility in several application fields
Cons	High instruments costs Bionformatic requirements	Sequencing cost are still higher than NGS Bionformatic requirements	No long reads Requires PCR amplification Does not allow direct RNA analysis Low accuracy in complex genomic regions analysis Time-consuming workflows

^1^ Data referred to the Revio system; ^2^ Data referred to PromethION 48, one flowcell; ^3^ Maximum read lengths in paired-end mode; ^4^ Data referred to the NovaSeq X Series; * Cost are extremely variable depending (for each technology) on the available platform, the used kits, and the application; moreover, costs may vary in different countries.
